# Transcriptome Analysis of Citrus Dwarfing Viroid Induced Dwarfing Phenotype of Sweet Orange on Trifoliate Orange Rootstock

**DOI:** 10.3390/microorganisms10061144

**Published:** 2022-06-01

**Authors:** Irene Lavagi-Craddock, Tyler Dang, Stacey Comstock, Fatima Osman, Sohrab Bodaghi, Georgios Vidalakis

**Affiliations:** 1 Department of Microbiology and Plant Pathology, University of California, Riverside, CA 92521, USA; irenela@ucr.edu (I.L.-C.); tdang004@ucr.edu (T.D.); scoms002@ucr.edu (S.C.); sohrab@ucr.edu (S.B.); 2 Department of Plant Pathology, University of California, Davis, CA 95616, USA; fmosman@ucdavis.edu

**Keywords:** RNA-seq, CDVd, transmissible small nuclear ribonucleic acid (TsnRNA), mRNA, functional analysis, differentially expressed genes (DEGs)

## Abstract

Dwarfed citrus trees for high-density plantings or mechanized production systems will be key for future sustainable citrus production. Citrus trees consist of two different species of scion and rootstock. Therefore, any observed phenotype results from gene expression in both species. Dwarfed sweet orange trees on trifoliate rootstock have been produced using citrus dwarfing viroid (CDVd). We performed RNA-seq transcriptome analysis of CDVd-infected stems and roots and compared them to non-infected controls. The identified differentially expressed genes validated with RT-qPCR corresponded to various physiological and developmental processes that could be associated with the dwarfing phenotype. For example, the transcription factors MYB13 and MADS-box, which regulate meristem functions and activate stress responses, were upregulated in the stems. Conversely, a calcium-dependent lipid-binding protein that regulates membrane transporters was downregulated in the roots. Most transcriptome reprogramming occurred in the scion rather than in the rootstock; this agrees with previous observations of CDVd affecting the growth of sweet orange stems while not affecting the trifoliate rootstock. Furthermore, the lack of alterations in the pathogen defense transcriptome supports the term “Transmissible small nuclear ribonucleic acid,” which describes CDVd as a modifying agent of tree performance with desirable agronomic traits rather than a disease-causing pathogen.

## 1. Introduction

Citrus (family *Rutaceae*) is an economically important crop with several high-value cultivars, including oranges, mandarins, grapefruits, and lemons. The citrus industry was recently valued at USD3.63 billion in California alone, with an estimated economic impact of USD7.6 billion [1]. Citrus fruits and flavors have a wide range of usage and are important sources of vitamins, antioxidants, minerals, and dietary fiber essential for overall nutritional wellbeing [2,3]. Citrus trees are produced by grafting the desired scion variety onto a suitable rootstock species and then planting it in commercial citrus orchards. Tree spacing in citrus orchards has reduced over time in favor of higher tree densities to maintain yield on the reduced available land for agriculture, increase economic returns, and combat the growing threat of citrus huanglongbing (HLB). However, high-density citrus plantings cannot be achieved without the use of dwarfed citrus trees [4,5,6,7].

Viroid infection can induce morphological and cytological changes that have been well documented for other plant and viroid species [8,9,10]. Viroid symptoms can include leaf epinasty, chlorosis, stunting, and reduced root mass. Other symptoms such as distorted cell walls, plasma membrane, mitochondria, and chloroplasts have also been observed [11,12,13]. In contrast, the citrus dwarfing viroid (CDVd) does not appear to cause major growth abnormalities and has been studied as a graft-transmissible dwarfing agent [14,15,16,17,18,19].

**Figure 1:**
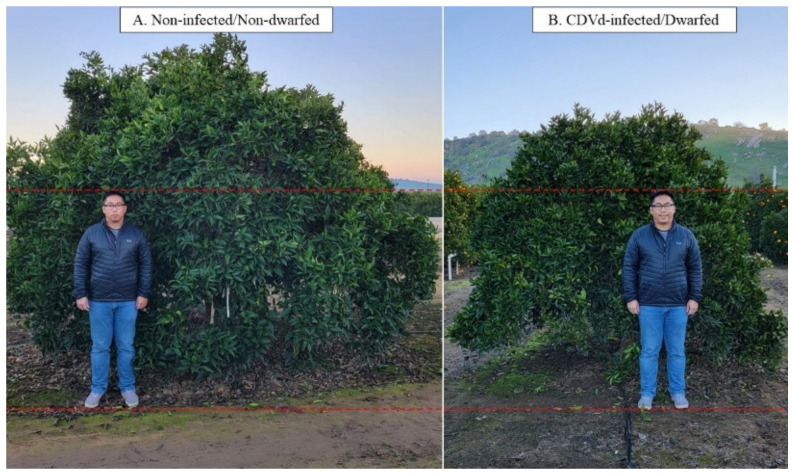
The observed dwarfing phenotype in navel orange (*Citrus sinensis* (L.) Osbeck) on trifoliate (*Citrus trifoliata* (L.) Raf.) rootstock. (**A**) is the non-infected/non-dwarfed tree and (**B**) is the citrus dwarfing viroid (CDVd)-infected/dwarfed tree.

In addition, CDVd-induced symptoms on citrus hosts depend on species, variety, and rootstock. CDVd infection of navel orange trees [*Citrus sinensis* (L.) Osbeck] propagated on trifoliate rootstock [*C. trifoliata* (L.), syn. *Poncirus trifoliata* (L.) Raf.] has shown that the observed stunting phenotype induced by CDVd-infection reduces canopy volume by approximately 50% (Figure 1), and the reduction in the canopy volume is a result of a >20% decrease in the apical growth of the shoots [19,20,21].

Therefore, there is a need to elucidate the molecular mechanism of the dwarfing phenotype, so the valuable information can be used to produce dwarfed trees for high-density plantings.

Currently, identifying potential targets of CDVd is challenging due to the nature of viroids, which do not encode for proteins, limiting the experimental approaches. In addition, most of the data on symptom induction by single viroids refer to experimental tests on the biological indicator ‘Etrog’ citron (*C. medica* L.). Previously, studies on CDVd-induced differentially expressed genes were performed on ‘Etrog’ citron in growth chamber conditions; microarray analysis identified mainly genes related to the cell wall structure, amino acid transport, signal transduction, and plant defense/stress response [22]. To this date, there are no transcriptome studies on mature dwarfed citrus field trees infected with CDVd [9].

To further explore the global effect of CDVd-infection on citrus host mRNAs and gain insight into the symptom development mechanism leading to the dwarfed phenotype observed in field plantings, we performed a transcriptome analysis of CDVd-infected dwarf citrus trees using a high throughput sequencing (HTS) approach. There has been limited transcriptomic research on citrus host response to viroid infection using the bioindicator ‘Etrog’ citron [23], while other viroid studies have used model plant systems such as *Solanum lycopersicum* and *Nicotiana tabacum* [11,24,25].

The improved availability of citrus genome and annotation has enabled the widespread use of HTS studies to develop a global understanding of the molecular machinery behind the response to pathogen infection [26]. Furthermore, HTS allows the discovery of rare transcripts, which would be difficult to identify using traditional methods such as microarrays, and is more time efficient than an RT-qPCR approach. More specifically, in citrus, HTS analysis of mRNA has been widely used to understand host responses to pathogens such as *‘Candidatus’* Liberibacter asiaticus [27,28,29,30] and citrus tristeza virus [28,31].

In this study, we performed a comparative analysis of differentially expressed genes in the stems and roots of CDVd-infected and non-infected sweet orange scion on trifoliate orange rootstock. This analysis provides valuable molecular information to understand the mechanisms responsible for the citrus dwarf phenotype in the field.

## 2. Materials and Methods

### 2.1. Plant Materials and RNA Isolation

Plant materials were collected in April 2016 from 18-year-old ‘Parent Washington’ navel orange on ‘Rich 16-6′ trifoliate orange trees. All trees were planted in the same east-west running orchard located at the University of California, Agriculture and Natural Resources, Lindcove Research and Extension Center (LREC) in Exeter, CA, USA. CDVd-infected trees were planted at high density (3 × 6.7 m), whereas non-infected control trees were spaced at standard density (6.1 × 6.7 m).

Stem and root samples were collected from the south side of the dwarfed CDVd-infected (*n* = 3) and the north and south sides of the full-size non-infected trees (*n* = 3). This type of sampling was necessary to represent the different sizes of trees (i.e., dwarfed trees 61.2% smaller than full size, [21]) and the different light distribution within their canopies due to the size and east-west orientation of the tree rows, in the transcriptome analysis.

After leaves and petioles were removed, the stems were roughly chopped into approximately 0.5–1 cm pieces, placed into 50 mL conical tubes, and flash-frozen with liquid nitrogen in the field. Feeder roots were sampled approximately 1 m away from the trunk and 20 cm deep, near the irrigation emitters, using a 10 cm diameter corer. The feeder root samples were washed thoroughly with water, gently blotted dry with paper towels, chopped into 0.5–1 cm pieces, placed into 50 mL conical tubes, and flash-frozen with liquid nitrogen in the field. Between each sample collection and processing, cutting tools and working surfaces were sanitized with 10% bleach solution (0.5–1% sodium hypochlorite) and then rinsed with water. New sterile disposable plasticware and razor blades were used to chop each sample. The frozen samples were transported to the Citrus Clonal Protection Program (CCPP), Citrus Diagnostic Therapy and Research Laboratory at the UC Riverside (Riverside, CA, USA) on dry ice and stored at −80 °C for downstream analysis (CDFA permit 3477).

Total RNA was isolated using the TRIzol^®^ reagent (Thermo Fisher Scientific, Waltham, MA, USA). For each sample, 300 mg of frozen tissue were ground in liquid nitrogen with sterilized mortar and pestle. The ground material was transferred to a 5 mL Eppendorf tube, and 3 mL of TRIzol^®^ reagent was added immediately. RNA extraction was performed according to the manufacturer’s instructions. The eluted RNA was aliquoted into four 1.5 mL microcentrifuge tubes to prevent freezing-thawing cycles during downstream analysis. The RNA concentration and quality were assessed with a spectrophotometer and the Agilent 2100 Bioanalyzer (Agilent, Santa Clara, CA, USA) using the Plant RNA Nano assay. The presence or absence of CDVd, as well as that of other graft-transmissible pathogens of citrus endemic to California, was confirmed in each sample by reverse transcription-quantitative polymerase chain reaction (RT-qPCR) as previously described [21,32].

### 2.2. Library Preparation and High Throughput Sequencing

Eighteen cDNA libraries were prepared for each sample using the Illumina TruSeq Stranded mRNA Kit (San Diego, CA, USA), following the manufacturer’s recommended protocol for Low Sample (LS) throughput. The libraries were quantified with the Agilent 2100 Bioanalyzer (Agilent, Santa Clara, CA, USA) using the High Sensitivity DNA Kit. The libraries were quantified using the in-house SeqMatic HT1 quantitative polymerase chain reaction (qPCR) assay (SeqMatic, Fremont, CA, USA) and pooled equimolar. The libraries were sequenced using an Illumina HiSeq^TM^ 4000 (San Diego, CA, USA) instrument with paired-end 100 bp reads (SeqMatic, Fremont, CA, USA). Raw reads were trimmed and demultiplexed for subsequent bioinformatic analysis.

### 2.3. Bioinformatic Analysis

The raw sequencing data of the north and south samples from each of the three non-infected full-size trees were concatenated into one file for analysis. Bioinformatic analyses were performed using the OmicsBox software suite version 2.0.36 (Cambridge, MA, USA) using the reference Valencia orange (version 1.0) (GCF_000317415.1) and trifoliate (v. 1.3.1) [33] genomes. RNA-seq alignment was performed using Spliced Transcript Alignment to a Reference (STAR v. 2.7.8a) [34] with default parameters. The quantification of transcript expression levels was performed using the HTSeq (v. 0.90) package [35], while the differential expression analysis was performed edgeR (v. 3.28.0) [36] with default parameters. Functional analysis was performed using the Blast2GO [37] tool within OmicsBox Suite. BLAST [38] was performed on the DEG sequences, and the gene ontology (GO) terms and Kyoto Encyclopedia of Genes and Genomes (KEGG) functions [39] were assigned. Protein functionality was confirmed with InterPro [40]. The dataset was uploaded into NCBI Sequence Read Archive under the accession numbers SAMN26677719 to SAMN26677722. All figures were created using GraphPad Prism version 9.3.0 (San Diego, CA, USA).

### 2.4. Expression Analysis of Citrus mRNA Target Genes Using RT-qPCR

RT-qPCR assays were designed to verify the expression levels of the predicted target genes (Supplementary Table S1). Actin was used as an internal control gene to determine the relative abundance of the target mRNA expression levels by the comparative Cq method. Reverse transcription was performed using the SuperScript^TM^ II Reverse Transcriptase (RT) (Thermo Fisher Scientific). The reactions were performed using the manufacturer’s recommended protocol as follows: 2 µL of oligo (dT) (500 µg/mL), 2 µL of dNTP (10 mM), 4 µL of total RNA (diluted to 100 µg/µL), and 16 µL of nuclease-free water with a final volume of 24 µL. All samples were standardized to the same concentration to ensure equal representation, and incubation steps were performed using the ProFlex thermal cycler (Thermo Fisher Scientific). The mixture was incubated for 5 min at 65 °C and subsequently chilled on ice. The first strand synthesis was prepared with 8 µL of 5x First-Strand Buffer, 4 µL of 0.1 M DTT, and 2 µL of RNaseOUT^TM^ (40 units/μL) (Thermo Fisher Scientific) and then incubated for 2 min at 42 °C. Finally, 2 µL of SuperScript^TM^ II RT (200 units) were added, and the reaction was incubated at 42 °C for 50 min followed by 15 min at 70 °C. Downstream qPCR was performed in triplicates, according to the MIQE guidelines [41], using the TaqMan^TM^ Fast Advanced Master Mix Kit (Thermo Fisher Scientific) as follows: 10μL of master mix, 8 μL of nuclease-free water, 1 μL of primer and probe mixture, and 1 μL of cDNA. FAM fluorophore was used for all qPCR probes. The qPCR was performed using the QuantStudio 12K Flex Real-Time PCR (Thermo Fisher Scientific) with the following conditions: 50 °C for 2 min, 95 °C for 2 min, and 95 °C for 1 s, 60 °C for 20 s for 40 cycles.

## 3. Results

### 3.1. Transcriptome Assembly and Annotation

To understand the citrus host transcriptome response to CDVd infection, we prepared and analyzed RNA-seq libraries from the stem and root samples of non-infected and CDVd-infected navel orange citrus trees on trifoliate orange rootstock. Overall, stems generated more reads than roots for non-infected and CDVd-infected trees (Table 1). A high percentage of reads (72–88%) from both the non-infected and CDVd-infected trees were mapped to their relative genomes using the STAR alignment software (Table 1). The average length of mapped reads in all samples tested was close to the expected size of 200 nt (Table 1).

### 3.2. Differentially Expressed Genes (DEGs) Analysis and Identification

**Figure 2:**
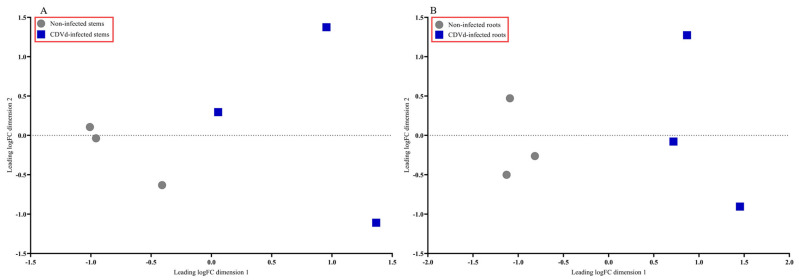
Multidimensional scaling (MDS) plot displaying similarity in leading log fold change in non-infected versus citrus dwarfing viroid (CDVd)-infected (**A**) stem and (**B**) root tissues.

The multidimensional scaling (MDS) plot showed distinct differences between the non-infected and CDVd-infected trees in stem and root tissues (Figure 2A,B). Non-infected stems and roots were similar and clustered closer together than the CDVd-infected tissues (Figure 2A,B).

**Figure 3:**
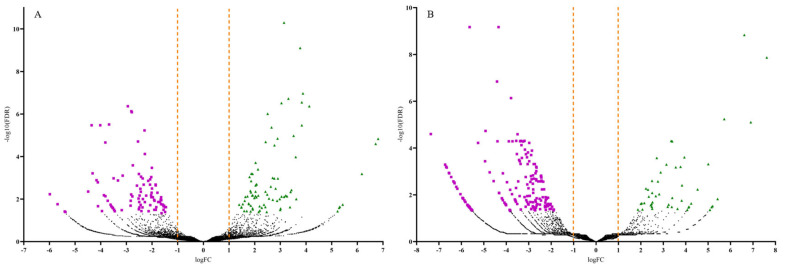
Volcano plots of the differentially expressed genes (DEGs) for both significantly upregulated and downregulated genes between non-infected and citrus dwarfing viroid (CDVd)-infected (**A**) stem and (**B**) root samples. Significantly upregulated (FDR < 0.05; logFC > 1.0) and downregulated (FDR < 0.05; logFC < −1.0) genes are represented in green (triangles) and magenta (squares), respectively.

Volcano plots were generated to display the number of up and downregulated DEGs by plotting the log fold change (FC) against the negative log (10)-transformed false discovery rate (FDR) values (Figure 3). Samples with high negative log (10)-transformed FDR values indicate DEGs with more significant regulation in response to CDVd-infection. Positive FC values indicated upregulated DEGs (FDR < 0.05; logFC > 1.0), while negative FC values indicated downregulated ones (FDR < 0.05; logFC < −1.0). Most upregulated DEGs were identified in the stems (83), whereas most of the downregulated DEGs were identified in the roots (186) (Figure 3 and Table 2).

**Figure 4:**
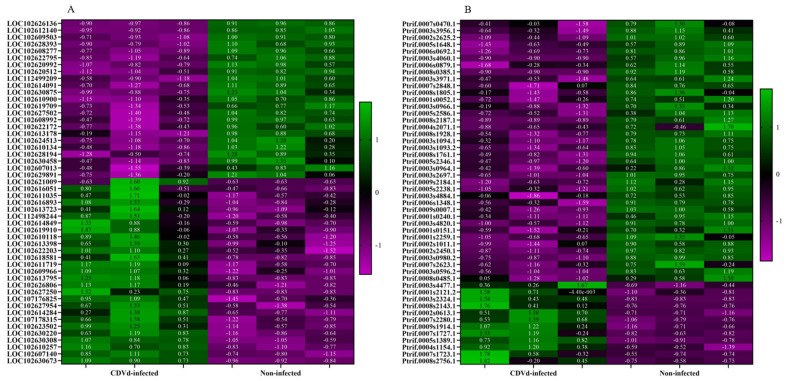
Heat map of the top 50 differentially expressed genes (DEGs) in both (**A**) stems and (**B**) roots ranked by FDR. The targets indicated in green are upregulated DEGs, while magenta targets are downregulated DEGs.

Heat maps of the top 50 DEGs identified from the stem and root tissues of non-infected and CDVd-infected trees were generated based on FDR (Figure 4). Results indicated a relatively even clustering of stem upregulated and downregulated DEGs in the non-infected and CDVd-infected samples. In contrast, the roots showed clustering skewed towards the DEGs that were significantly downregulated in the CDVd-infected and upregulated in the non-infected samples (Figure 4A,B).

DEG analysis identified up- and downregulated targets. In the stem, the probable 28S rRNA (cytosine-C(5))-methyltransferase (FDR = 5.05 × 10^−11^; logFC = 8.83) and the pentatricopeptide repeat-containing protein (FDR = 4.29 × 10^−7^; logFC = −2.95) had the most statistically significant fold change (Table 3).

In the roots, the plant invertase/pectin methylesterase inhibitor superfamily protein (FDR = 2.48 × 10^−04^; logFC 3.94) and the kinase-inducible domain interacting 9, Kix9 (FDR = 6.75 × 10^−10^; logFC = −5.64) had the most statistically significant fold change (Table 4).

Further analysis showed that the highest logFC among the upregulated stem DEGs corresponded to the dihydrolipoyllysine-residue acetyltransferase component 5 of pyruvate dehydrogenase complex, chloroplastic (LOC102627250) (logFC = 6.81; FDR = 1.47 × 10^−5^), while the highest logFC for downregulated stem DEGs corresponded to the pentatricopeptide repeat-containing protein, mitochondrial-like (LOC107176200) (logFC = −5.98; FDR 0.005).

The highest logFC for the upregulated root DEGs corresponded to the HSP20-like chaperone super family protein (Ptrif.0003s2324.1) (logFC 7.61; FDR 1.35E−08), and the highest logFC for the downregulated root DEGs corresponded to the calcium-dependent lipid-binding (CaLB domain) family protein (Ptrif.0003s4060.1) (logFC −7.3; FDR 2.53 × 10^−5^). The complete list of all DEGs identified in this study can be found in Supplementary Tables S2–S5.

### 3.3. Confirmation of Candidate DEGs by RT-qPCR Analysis

**Figure 5:**
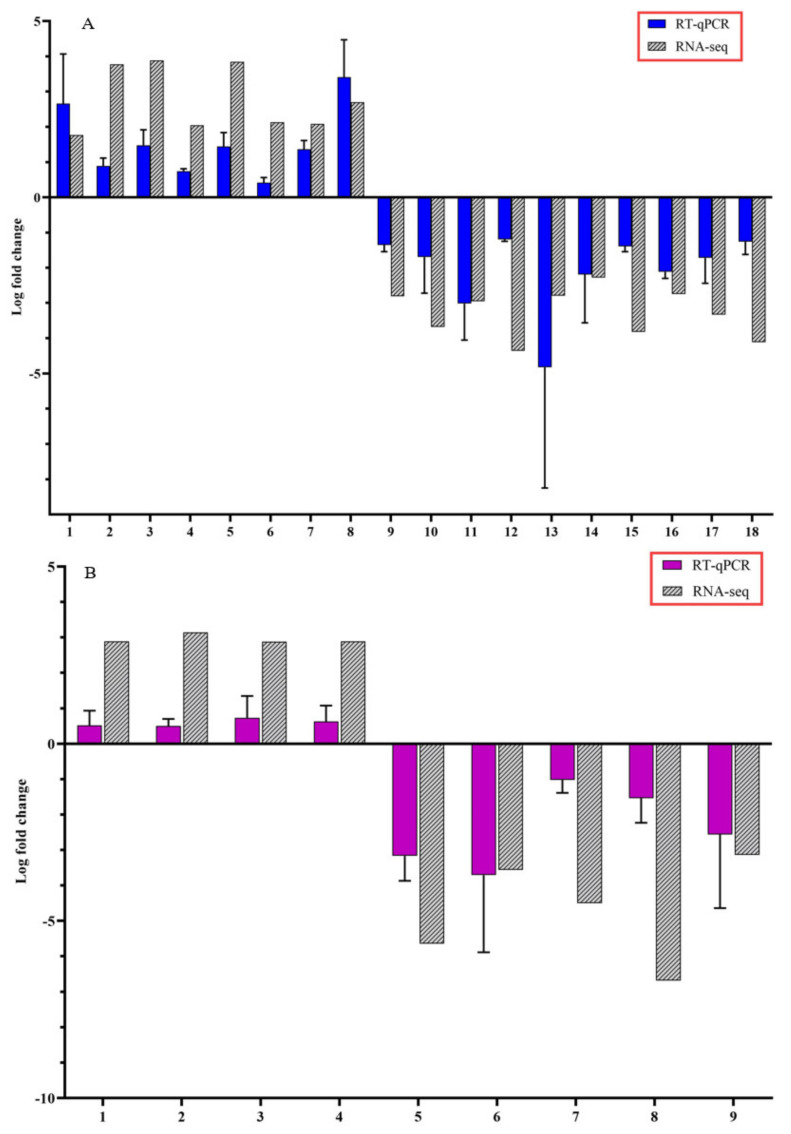
Expression pattern validation of selected candidate differentially expressed genes (DEGs) by reverse transcription-quantitative polymerase chain reaction (RT-qPCR) on stems and roots of citrus dwarfing viroid (CDVd) infected relative to non-infected citrus trees. The relative gene expression was evaluated by the comparative Cq method using actin as a reference gene. The targeted DEGs used in the analysis for stems (**A**) were: (1) receptor-like protein 9DC3 (LOC102630240); (2) germin-like protein subfamily T member (LOC102611719); (3) pentatricopeptide repeat-containing protein At2g17525 (LOC102610118); (4) MADS-box transcription factor 23-like (LOC102630220); (5) phospholipase A1-Ibeta2 (LOC102609966); (6) protein TIC 55 (LOC102614849); (7) pentatricopeptide repeat-containing protein At1g08070 (LOC102619843); (8) transcription factor MYB13 (LOC102616893); (9) SKP1-like protein 21 (LOC102620512); (10) amino-acid permease BAT1-like (LOC102620992); (11) LOC102627502 pentatricopeptide repeat-containing protein At4g21300; (12) uncharacterized protein (LOC102608277); (13) protein SHORT-ROOT-like (LOC102610900); (14) protein IQ-DOMAIN 1, transcript variant X1 (LOC102628194); (15) hydroxyproline O-galactosyltransferase GALT3, transcript variant X3 (LOC102626136); (16) cytochrome P450 82C4-like; (17) LOC102622795 thaumatin-like protein 1 (LOC102619709); (18) Plant uncharacterized (LOC102625692). Targeted DEGs used in the analysis for the roots (**B**) were: (1) Plant invertase/pectin methylesterase inhibitor superfamily protein (Ptrif.0009s1914.1); (2) S-adenosyl-L-methionine-dependent O-methyltransferase/ethylene-responsive transcription factor ERF114 (Ptrif.0005s1389.1); (3) C2H2-like zinc finger protein (Ptrif.0005s2465.1); (4) Pentatricopeptide repeat (PPR) superfamily protein (Ptrif.0006s1595.1); (5) Kinase-inducible domain interacting 9 (Ptrif.0001s0240.1); (6) Pentatricopeptide repeat (PPR-like) superfamily protein (Ptrif.0003s3971.1); (7) zinc-finger protein 10 (Ptrif.0006s0692.1); (8) Calcium-dependent lipid-binding (CaLB domain) family protein (Ptrif.0003s4060.1); (9) GNS1/SUR4 membrane protein family (Ptrif.0005s2586.1). Error bars for the RT-qPCR are standard deviations.

To confirm the accuracy of Illumina RNA-seq results, RT-qPCR was performed on selected candidate DEGs. The logFC values of the RNA-seq and RT-qPCR were consistent and showed similar trends for both up- and downregulated stem and root DEGs (Figure 5).

### 3.4. Functional Classification of DEGs

**Figure 6:**
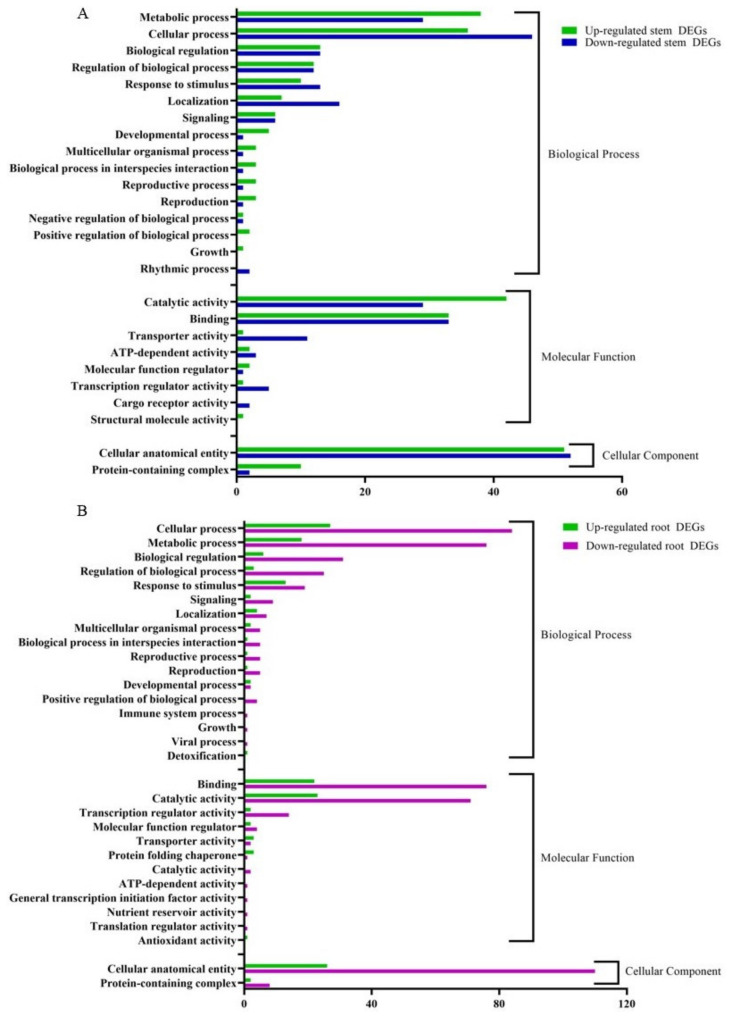
Clusters of orthologous groups functional classification of differentially expressed genes (DEGs), between non-infected and citrus dwarfing viroid (CDVd)-infected (**A**) stem and (**B**) root tissues. The bar graph shows the number of sequences and distribution in different functional categories of the predicted targets at gene ontology (GO) level 2.

The RNA-seq data were annotated based on the gene ontology (GO) terms, which categorized the DEGs in response to CDVd infection for both stems and roots into various biological, molecular, and cellular processes (Figure 6). Among the upregulated DEGs in the stems, there were 143 associated with biological process (BP), 82 with molecular function (MF), and 614 with cellular component (CC). Within the downregulated DEGs in the stems, 143 were associated with BP, 84 with MF, and 54 with CC. The top stem DEGs categorized in BP were (i) metabolic process (up 38 and down 29); (ii) cellular process (up 36 and down 46) (iii) Biological regulation (up 13 and down 12). For stem DEGs associated with MP (i) catalytic activity (up 42 and down 29); (ii) binding (up and down 33); (iii) transport activity (down 11 and up 1). Stem DEGs that belong to the CC were (i) cellular anatomical entities (up 51 and down 52) and (ii) protein-containing complexes (up 10 and down 2) (Figure 6A). A list of all GO term annotations for the stem DEGs can be found in Supplementary Tables S6 and S7.

From the upregulated DEGs identified in the roots, there were 81 associated with BP, 56 with MF, and 28 CC. Among the downregulated root DEGs, 280 were associated with BP, 174 with MF, and 118 CC. The top root DEGs in BP were categorized into (i) cellular process (up 27 and down 84), (ii) metabolic process (up 18 and down 76), and (iii) Biological regulation (up 6 and down 31). The top root DEGs in MF were associated with (i) binding (up 22 and down 76); (ii) catalytic activity (up 23 and down 71); (iii) transcription regulatory activity (up 2 and down 14). Only two GO terms were associated with CC (i) cellar anatomical entity (up 26 and down 110) and (ii) protein-containing complex (up 2 and down 8) (Figure 6B). A list of all GO term annotations for the root DEGs can be found in Supplementary Tables S8 and S9.

**Figure 7:**
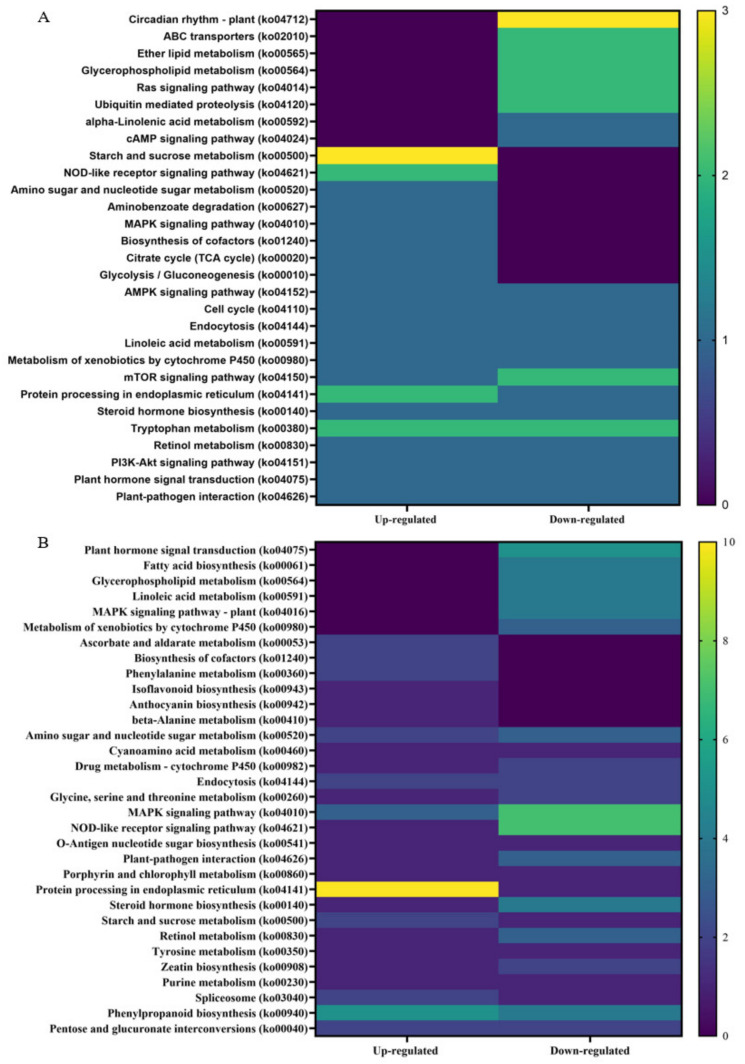
Distribution of Kyoto Encyclopedia of Genes and Genomes (KEGG) pathway classification of upregulated differentially expressed genes (DEGs) between non-infected and citrus dwarfing viroid (CDVd)-infected (**A**) stem and (**B**) root tissues. The heat map shows the KEGG function and the number of associated sequences.

Kyoto Encyclopedia of Genes and Genome (KEGG) analysis was performed on up- and downregulated DEGs from non-infected and CDVd-infected trees from the stem and root tissues (Figure 7). The KEGG results for stem upregulated DEGs identified 82 pathways and 25 sequences linked to specific pathways. The pathways with the most sequences from upregulated stem DEGs were starch and sucrose metabolism (3) (ko00500), NOD-like receptor signaling pathway (2) (ko04621), tryptophan metabolism (2) (ko00980), and protein processing in the endoplasmic reticulum (2) (ko04141). KEGG analysis also identified 96 pathways and 25 sequences in stem downregulated DEGs, with the highest downregulated pathway being the plant circadian rhythm (3) (ko04712) (Figure 7A). KEGG analysis for roots upregulated DEGs found 73 pathways and 26 sequences and 118 pathways and 53 sequences from downregulated roots. The highest number of sequences associated with upregulated roots were protein processing in the endoplasmic reticulum (10 sequences) (ko04141). In contrast to the downregulated roots, the NOD-like receptor signaling pathway (ko04621) (7), MAPK signaling pathway (7) (ko04010), and plant hormone signal transduction (5) (ko04075) were the highest (Figure 7B). A complete list of all KEGG results can be found in Supplementary Tables S10–S13.

## 4. Discussion

Citrus is among the most economically valuable fruit crops. In 2019, citrus production exceeded 157.9 million tons in over 9.8 million hectares worldwide [1]. The ever-reducing global natural resources (i.e., decreased arable land and water availability) combined with climate change, agricultural labor shortages, and the increased disease pressure of deadly citrus diseases such as HLB demand the adoption of novel citriculture practices. High-density citrus plantings, the possible implementation of mechanized citrus harvest, and citrus production under protective structures are some of the most promising trends for sustainable citriculture. The ability to produce dwarf citrus trees is critical for implementing these innovations and maximizing returns on investments [19,42,43,44,45,46].

Dwarfed sweet orange trees propagated on trifoliate orange rootstock, where reduced vegetative growth is achieved without compromising fruit yield or quality, have been produced using CDVd. CDVd is part of a group of so-called “graft-transmissible dwarfing agents,” the use of which has been explored for almost fifty years since it was initially proposed [16,18,47,48,49,50]. Previously, we observed CDVd significantly reduced sweet orange canopy volume on trifoliate rootstock by reducing vegetative growth [19,20,21]. Long-term field observations indicated that CDVd might be used as a possible tool for high-density plantings of citrus. In addition, key findings on the possible biological mechanism through which CDVd affects specific rootstock scion combinations to reduce tree canopy volume were obtained [14]. Understanding the detailed molecular mechanisms leading to reduced canopy volume of commercial citrus tree varieties in response to CDVd infection is critical. Indeed, it would provide the information to produce reduced-size citrus trees without a graft-transmissible viroid agent.

Transcriptomic profiling provides a rapid and cost-effective approach to identifying target genes responsible for the observed reduced vegetative growth of CDVd-infected sweet orange trees. Commercial field citrus trees consist of two different species: the scion, producing the canopy of the tree (i.e., branches, stems, and leaves), and the rootstock, from which the root system of the tree (i.e., taproot, lateral, and feeder roots) develops [51]; therefore, any observed tree phenotype is a result of gene expression not in one but two species that closely interact with each other. In this study, we performed a transcriptome analysis of CDVd-infected stems and roots from sweet orange on trifoliate rootstock trees grown in high-density plantings and compared them with non–infected controls. Here, we showed that CDVd infection affects a wide range of biological functions via different stem and root mRNA transcripts. Furthermore, it complements recent molecular studies targeting CDVd-derived small RNAs on dwarfed citrus [32]. As a result of differential expression analysis, a total of 409 CDVd-dependent differentially expressed genes (DEGs) were deregulated (131 upregulated and 278 downregulated), with the majority of upregulated genes in the stems (83/131), and most downregulated genes in the roots (186/278) (Table 2).

The five most significant upregulated genes in CDVd-infected stems include: LOC102607140, a probable 28S rRNA (cytosine-C(5))-methyltransferase, an ortholog of the *Arabidopsis thaliana* (AT5G26180), which plays a role in regulating translation in response to stress [52]; LOC102611719, a germin-like protein subfamily T member. Germin-like proteins (GLPs) are encoded by multigene families in several plant species, and some subfamily members play a role in defense against pathogen attack [53,54]; LOC102610118, a pentatricopeptide repeat-containing protein (ortholog of AT2g17525). Pentatricopeptide repeat (PPR) proteins belong to a large gene family in plants. Some PPR proteins play important roles in organellar RNA metabolism and organ development in Arabidopsis and rice, while their functions remain unknown in woody species [55]; LOC102616051, cytosolic sulfotransferase 15-like, an ortholog of the Arabidopsis cytosolic sulfotransferase genes. In Arabidopsis, a hydroxyjasmonate sulfotransferase was reported to participate in the hydroxylation and sulfonation reactions that might be components of a pathway that inactivates excess jasmonic acid in plants [56]. The LOC102614284 is associated with aspartic proteinase Asp1-like, and plant aspartic proteinases have been implicated in diverse cellular functions, including protein processing and/or degradation, plant senescence, stress responses, programmed cell death, and reproduction [57].

The five most significantly downregulated genes in CDVd-infected stems include: LOC102627502, a pentatricopeptide repeat-containing protein, an ortholog of At4g21300; LOC102620512, SKP1-like protein 21, which is involved in ubiquitination and subsequent proteasomal degradation of target proteins [58]; LOC102610900, SHORT-ROOT-like protein, which controls radial patterning of the Arabidopsis root, and therefore root growth [59], which is consistent with reduced root mass in viroid infected plants [9]; LOC102620992, an amino-acid permease BAT1-like, which plays a role in primary carbon metabolism and plant growth by mediating the transport of gamma amino butyric acid from the cytosol to mitochondria in Arabidopsis [60]; LOC102628393, serine/threonine-protein kinase ATG1a, involved in autophagy in a nutritional condition-dependent manner [61].

In CDVd-infected roots, the five most significantly upregulated genes include: Ptrif.0009s1914.1 (ortholog of AT5G62350/LOC_Os06g49760), a plant invertase/pectin methylesterase inhibitor superfamily protein, which inhibits pectin methylesterases (PMEs) and invertases (PMIs) and has been implicated in the regulation of fruit development, carbohydrate metabolism, and cell wall extension as well as inhibition of microbial pathogen PMEs. The interplay between PME and PMEI is a determinant of cell adhesion, cell wall porosity, and elasticity, as well as a source of signaling molecules released upon cell wall stress [62]. The Ptrif.0004s1154.1 (ortholog of AT5G07310/Cre14.g620500/LOC_Os04g32620) is an integrase-type DNA-binding superfamily protein involved in the regulation of transcription and recently reported to integrate the jasmonate and cytokinin signaling pathways to repress adventitious rooting in Arabidopsis [63]; Ptrif.0005s1389.1, an ortholog of Cre05.g240400/LOC_Os12g25450, S-adenosyl-L-methionine-dependent O-methyltransferase/ethylene-responsive transcription factor, which methylates proteins, small molecules, lipids, and nucleic acids [64]; Ptrif.0005s2465.1 (ortholog of AT5G52010/LOC_Os06g07020) C2H2-like zinc finger protein, which contains one of the most common domains found in the transcription factors of higher eukaryotes [65]; and Ptrif.0006s1595.1 (ortholog of AT5G03800) another pentatricopeptide repeat (PPR) superfamily protein.

The five most significantly downregulated genes in CDVd-infected roots include: Ptrif.0001s0240.1 (ortholog of AT4G32295), which in Arabidopsis is part of a transcriptional repressor complex that regulates leaf growth [66]; Ptrif.0003s0980.2, (ortholog of LOC_Os04g48160) a putative IQ calmodulin-binding motif family protein; Ptrif.0006s0692.1, an ortholog of AT2G37740/LOC_Os05g20930, zinc-finger protein 10, a transcription factor that may regulate cell division and growth [67]; Ptrif.0003s4060.1 (ortholog of AT4G01200) a calcium-dependent lipid-binding (CaLB domain) family protein, a repressor of abiotic stress responses in Arabidopsis [68]; and Ptrif.0005s2586.1 (ortholog of AT1G75000/LOC_Os12g43890) a GNS1/SUR4 membrane protein family, enzymes related to very long-chain fatty acid synthesis in Arabidopsis [69].

Among the additional interesting CDVd-responsive stem genes whose differential expression was verified by RT-qPCR (Figure 5A), several were found including the plant defense receptor-like protein 9DC3 (LOC102630240) [70]; the MADS-box transcription factor 23-like (LOC102630220) [71], which is part of the transcription factor (TF) families (WRKY, MADS-box and MYB) that activate unique abiotic and biotic stress-responsive strategies considered as key determinants for defense and developmental processes in most eukaryotic plants [72]; the phospholipase A1-Ibeta2 (LOC102609966) [73], the chloroplast import protein TIC 55 (LOC102614849), the chloroplast import protein [74]; the chloroplast pentatricopeptide repeat-containing protein ortholog of At1g08070 (LOC102619843), which is involved in RNA editing events within the chloroplast [75]; the transcription factor MYB13 (LOC102616893), which regulates meristem function by being a component of a regulatory network controlling the establishment and/or development of the shoot system in Arabidopsis [76]; protein IQ-DOMAIN 1, transcript variant X1 (LOC102628194) which plays a role with calmodulins or calmodulin-like proteins as well as involved in scaffolding in cellular signaling and trafficking [77,78]; hydroxyproline O-galactosyltransferase GALT3, transcript variant X3 (LOC102626136), which commits arabinogalactan proteins to the first step in arabinogalactan polysaccharide addition. AGP glycans play essential roles in both vegetative and reproductive plant growth [79]; cytochrome P450 82C4-like, which is involved in the early Fe deficiency response in Arabidopsis [80]; LOC102622795; thaumatin-like protein 1 (LOC102619709), involved in local responses of roots to colonization by non-pathogenic plant growth-promoting rhizobacteria [81].

In roots, CDVd-responsive genes verified by RT-qPCR included integrase-type DNA-binding superfamily protein (Ptrif0004s1154.1), involved in transcriptional regulation S-adenosyl-L-methionine-dependent O-methyltransferase/ethylene-responsive transcription factor ERF114 (Ptrif.0005s1389.1), involved in shoot development and architecture in Arabidopsis [82,83]; and the zinc-finger protein 10 (Ptrif.0006s0692.1), the overexpression of which was associated with dwarf plants in Arabidopsis [67]. Calcium-dependent lipid-binding (CaLB domain) family protein (Ptrif.0003s4060.1). Stress affects cell ion homeostasis, and plants adjust it by regulating membrane transporters and channels. Ca2+ is key in such a process as it regulates the protein kinases and phosphatases that control ion transport activity in response to environmental stimuli [84] (Supplementary Table S5).

Gene ontology (GO) assignments were used to classify the function of the identified DEGs. GO term enrichment analysis showed that in both stems and roots of CDVd-infected trees, for the biological process category, the GO terms corresponding to metabolic process and cellular process were the most enriched terms in response to CDVd; for molecular function, the catalytic activity and binding are the most enriched, and for the cellular component category, the cellular anatomical entity term was most enriched in the stems (Figure 6A,B).

In order to gain further insight into the function of DEGs correlating to a reduced vegetative growth as observed in CDVd-infected trees, the KEGG pathway classification was performed. Overall, circadian rhythms (ko04712), starch and sucrose metabolism (ko00500), and protein processing in the endoplasmic reticulum (ko04141) were most altered in response to CDVd infection. Circadian rhythms (ko04712), downregulated in CDVd-infected stems, are known to cross-talk with defense signaling against bacteria in plants [85] and to be important determinants in the outcome of plant-pathogen interactions [86]. Starch and sucrose metabolism (ko00500) were upregulated in both CDVd-infected stems and roots. Sucrose, the main form of assimilated carbon produced during photosynthesis, has important roles as a signaling molecule, and it is involved in many metabolic processes in plants; it is essential for plant growth and development and plays a role in plant defense by activating plant immune responses against pathogens. Upon infection, pathogens hijack the plant metabolism to access the plant sugars and, in doing so, trigger plant defense responses. Invertases appear to be involved in establishing plant defense responses [87].

Protein processing in the endoplasmic reticulum (ko04141) was upregulated in roots and stems, consistent with higher metabolic activity levels. Tryptophan metabolism (ko00380) was up- and downregulated in stems. Tyrosine and tryptophan are precursors for the plant defense compounds dhurrin and indole glucosinolates, respectively. In addition, tryptophan is a precursor for the essential phytohormone indole-3-acetic acid [88]. Other KEGG pathway classification terms that were altered in response to CDVd infection include: Lipid metabolism (glycerophospholipid (ko00564), which was downregulated in roots and stems; ether lipid (ko00565) was downregulated in stems; ABC transporters (ko 02010), Ras signaling pathway, mTOR signaling pathway (ko04150), which were downregulated in stems.

To date, only one previous citrus gene expression profiling study in response to CDVd infection has been reported [22], and it differs from ours in many important aspects. Indeed, it predates the widespread use of HTS applications and the availability of reference genomes for *C. sinensis* and *P. trifoliata*. Instead, the authors profiled the transcriptome using the differential display technique (DDRT-PCR). Most importantly, however, the authors studied the sensitive viroid bioindicator ‘Etrog’ citron, Arizona 861-S1, newly inoculated with CDVd (18 months old) grown under growth chamber conditions and expressing symptoms of leaf drooping and petiole necrosis. Provided these experimental conditions, as expected, among the upregulated genes, a suppressor of RNA silencing was identified [22]. In contrast, our study used HTS technologies to analyze 18-year-old, field-grown commercial citrus tree varieties that do not express typical viroid symptoms of leaf epinasty and midvein and petiole necrosis [89]. Therefore, it is not surprising that the transcriptome profiling was different.

When taken together, our results indicate that CDVd modulates the expression profile of important citrus growth and developmental processes that may participate in the cellular changes leading to the observed phenotype of reduced vegetative growth and overall smaller tree size. In this study, most of the transcriptome expression reprogramming appeared to occur in the stems (*C. sinensis*) rather than in the roots (*C. trifoliata*), which is consistent with the phenotypic observation of the reduced stem vegetative growth of the stems and striking dwarfed citrus tree phenotype in CDVd-infected trees [21]. It is also in agreement with the observation that the trifoliate orange rootstock does not display major symptoms in response to CDVd infection [90,91,92]. Even though our study demonstrates the potential use of modern molecular technologies to decipher complex plant responses to biotic factors using plants growing in agricultural production systems, future studies employing transgenics and model plant systems could provide additional evidence to dissect further the genes and pathways participating in the development of the dwarf phenotype identified in this study. 

Lastly, the lack of major alterations in the pathogen defense transcriptome profile in our study is in contrast to studies with other viroids that demonstrated altered expression of plant defense genes [11,24]. This finding supports the idea that when viroid RNAs such as CDVd are used for horticultural purposes, such as reducing tree canopy volume, perhaps they are better described as “Transmissible small nuclear ribonucleic acids” (TsnRNAs). This term provides a more detailed description of the TsnRNA’s hallmark properties (i.e., transmissibility, small genome size, site of replication, and RNA nature) as well as dissociation from a generic virology term, such as “viroid”, which implies disease and crop loss. Therefore TsnRNAs that do not express a disease syndrome but rather act as modifying agents of tree performance resulting in desirable agronomic traits, such as reduced tree size for high-density plantings with potential economic and sustainability advantages, can be distinguished from the pathogenic viroids [18,20].

## Figures and Tables

**Table 1:** Summary of sequencing and STAR alignment results for non-infected and citrus dwarfing viroid (CDVd)-infected trees from the stem (*Citrus sinensis* (L.) Osbeck) and root tissues (*C. trifoliata* (L.).

Treatment	Avg. Total Reads	Avg. Uniquely Mapped Reads	Avg. Percent Mapped Reads	Avg. Mapped Length
Non-infected stem	57,720,333 ± 7,556,534	40,055,889 ± 10,591,488	71.63%	199.04 ± 0.25
CDVd-infected stem	36,254,830 ± 15,692,505	31,769,508 ± 13,584,944	87.87%	199.51 ± 0.32
Non-infected root	58,030,198 ± 9,821,953	48,928,990 ± 10,744,466	83.95%	199.8 ± 0.2
CDVd-infected roots	25,671,752 ± 1,148,290	22,553,155 ± 1,891,667	87.78%	199.98 ± 0.09

**Table 2:** Number of upregulated and downregulated differentially expressed genes identified in stem (*Citrus sinensis* (L.) Osbeck) and root tissues (*C. trifoliata* (L.)).

Tissue Type	Upregulated	Downregulated	Total
Stem	83	92	175
Root	48	186	234

**Table 3:** List of top 5 up and downregulated differentially expressed genes (DEGs) in non-infected vs. citrus dwarfing viroid (CDVd)-infected for stem tissues based on the FDR.

	Accession	Product	Fold Change (FC)	LogFC	Log Count Per Million (CPM)	*p*-Value	FDR
**Upregulated stem**	LOC102607140	probable 28S rRNA (cytosine-C(5))-methyltransferase	8.83	3.14	11.04	2.70 × 10^−15^	5.05 × 10^−11^
	LOC102611719	germin-like protein subfamily T member	13.66	3.77	7.41	8.39 × 10^−14^	7.84 × 10^−10^
	LOC102610118	pentatricopeptide repeat-containing protein At2g17525	14.72	3.88	6.52	1.73 × 10^−11^	1.08 × 10^−7^
	LOC102616051	cytosolic sulfotransferase 15-like	17.47	4.13	6.04	1.62 × 10^−10^	4.29 × 10^−7^
	LOC102614284	aspartic proteinase Asp1-like	5.67	2.50	9.39	5.71 × 10^−10^	9.70 × 10^−7^
**Downregulated stem**	LOC102627502	pentatricopeptide repeat-containing protein At4g21300	−7.70	−2.95	7.86	1.84 × 10^−10^	4.29 × 10^−7^
	LOC102620512	SKP1-like protein 21	−7.01	−2.81	8.07	3.56 × 10^−10^	7.40 × 10^−7^
	LOC102610900	protein SHORT-ROOT-like	−6.90	−2.79	8.06	4.41 × 10^−10^	8.24 × 10^−7^
	LOC102620992	amino-acid permease BAT1-like	−12.78	−3.68	6.34	1.99 × 10^−9^	3.11 × 10^−6^
	LOC102628393	serine/threonine-protein kinase ATG1a	−16.21	−4.02	6.03	2.69 × 10^−9^	3.38 × 10^−6^

**Table 4:** List of top 5 up and downregulated differentially expressed genes (DEGs) in non-infected vs. citrus dwarfing viroid (CDVd)-infected for roots tissues based on the FDR.

	Gene ID	Alt Gene ID	Product	Fold Change (FC)	LogFC	Log Count Per Million (CPM)	*p*-Value	FDR
**Upregulated roots**	Ptrif.0009s1914.1	AT5G62350/LOC_Os06g49760	Plant invertase/pectin methylesterase inhibitor superfamily protein	15.33	3.94	7.00	3.62 × 10^−7^	2.48 × 10^−4^
	Ptrif.0004s1154.1	AT5G07310/Cre14.g620500/LOC_Os04g32620	Integrase-type DNA-binding superfamily protein	6.54	2.71	12.63	4.04 × 10^−7^	2.64 × 10^−4^
	Ptrif.0005s1389.1	Cre05.g240400/LOC_Os12g25450	S-adenosyl-L-methionine-dependent O-methyltransferase/ethylene-responsive transcription factor ERF114	8.82	3.14	7.80	1.02 × 10^−6^	5.05 × 10^−4^
	Ptrif.0005s2465.1	AT5G52010/LOC_Os06g07020	C2H2-like zinc finger protein	7.38	2.88	8.03	2.69 × 10^−6^	1.09 × 10^−3^
	Ptrif.0006s1595.1	AT5G03800	Pentatricopeptide repeat (PPR) superfamily protein	7.43	2.89	8.06	2.73 × 10^−6^	1.09 × 10^−3^
**Downregulated roots**	Ptrif.0001s0240.1	AT4G32295	Kinase-inducible domain interacting 9, Kix9	−49.72	−5.64	9.20	6.04 × 10^−14^	6.75 × 10^−10^
	Ptrif.0003s0980.2	LOC_Os04g48160	IQ calmodulin-binding motif family protein, putative, expressed	−13.83	−3.79	10.36	1.96 × 10^−10^	7.31 × 10^−7^
	Ptrif.0006s0692.1	AT2G37740/LOC_Os05g20930	zinc-finger protein 10	−30.39	−4.93	7.76	7.47 × 10^−9^	1.85 × 10^−05^
	Ptrif.0003s4060.1	AT4G01200	Calcium-dependent lipid-binding (CaLB domain) family protein	−164.81	−7.36	7.11	1.13 × 10^−8^	2.53 × 10^−5^
	Ptrif.0005s2586.1	AT1G75000/LOC_Os12g43890	GNS1/SUR4 membrane protein family	−11.31	−3.50	9.35	1.27 × 10^−8^	2.58 × 10^−5^

## Data Availability

The dataset presented in this study can be found in NCBI Sequence Read Archive under the accession numbers SAMN26677719 to SAMN26677722.

## Supplementary Materials

The following are available online at: https://www.mdpi.com/article/10.3390/microorganisms10061144/s1, Table S1: List of primers used for RT-qPCR relative quantification of mRNA target genes; Table S2: List of upregulated DEGs identified in stem (*Citrus sinensis* (L.) Osbeck) tissue; Table S3: List of downregulated DEGs identified in stem (*Citrus sinensis* (L.) Osbeck) tissue; Table S4: List of upregulated DEGs identified in root (*Citrus trifoliata* (L.)) tissue; Table S5: List of downregulated DEGs identified in root (*Citrus trifoliata* (L.)) tissue; Table S6: List of GO terms for upregulated DEGs identified in stem (*Citrus sinensis* (L.) Osbeck) tissue; Table S7: List of GO terms for downregulated DEGs identified in stem (*Citrus sinensis* (L.) Osbeck) tissue; Table S8: List of GO terms for upregulated DEGs identified in root (*Citrus trifoliata* (L.)) tissue; Table S9: List of GO terms for downregulated DEGs identified in root (*Citrus trifoliata* (L.)) tissue; Table S10: List of KEGG for upregulated DEGs identified in stem (*Citrus sinensis* (L.) Osbeck) tissue; Table S11: List of KEGG for downregulated DEGs identified in stem (*Citrus sinensis* (L.) Osbeck) tissue; Table S12: List of KEGG for upregulated DEGs identified in root (*Citrus trifoliata* (L.)) tissue; Table S13: List of KEGG for downregulated DEGs identified in root (*Citrus trifoliata* (L.)) tissue.
